# The Instinct of Nursing

**Published:** 1879-08

**Authors:** 


### THE INSTINCT OF NURSING.'

Never feed infants with a spoon or allow them to drink their food, *as a rule.* Nature intends that the infant shall take food by sucking it into the mouth. The act of sucking causes the saliva to be poured out, to mingle with the food and aid in its digestion. An infant that loses the instinct of sucking, or who fails to exercise it as nature dictates, is doomed to invalidity and, probably, an early death.

